# Binary Lead Fluoride Pb_3_F_8_

**DOI:** 10.1002/chem.201903954

**Published:** 2019-11-04

**Authors:** H. Lars Deubner, Malte Sachs, Jascha Bandemehr, Sergei I. Ivlev, Antti J. Karttunen, Stefan R. Kachel, Benedikt P. Klein, Lukas Ruppenthal, Maik Schöniger, Claudio K. Krug, Jan Herritsch, J. Michael Gottfried, Jamal N. M. Aman, Jörn Schmedt auf der Günne, Florian Kraus

**Affiliations:** ^1^ Fachbereich Chemie Philipps-Universität Marburg Hans-Meerwein-Str. 4 35032 Marburg Germany; ^2^ Department of Chemistry and Materials Science Aalto University 00076 Aalto Finland; ^3^ Inorganic Materials Chemistry University of Siegen Adolf-Reichwein-Str. 2 57076 Siegen Germany

**Keywords:** IR and Raman spectroscopy, lead fluoride, NEXAFS, NMR spectroscopy, quantum chemical calculations

## Abstract

The binary lead fluoride Pb_3_F_8_ was synthesized by the reaction of anhydrous HF with Pb_3_O_4_ or by the reaction of BrF_3_ with PbF_2_. The compound was characterized by single‐crystal and powder X‐ray diffraction, IR, Raman, and solid‐state MAS ^19^F NMR spectroscopy, as well as thermogravimetric analysis, XP and near‐edge X‐ray absorption fine structure (NEXAFS) spectroscopy. Solid‐state quantum‐chemical calculations are provided for the vibrational analyses and band assignments. The electronic band structure offers an inside view of the mixed valence compound.

## Graphical abstract

**A binary lead(II/IV) fluoride**: Binary lead fluoride Pb_3_F_8_ was synthesized by the reaction of anhydrous HF with Pb_3_O_4_ or by the reaction of BrF_3_ with PbF_2_ (see figure) The compound was characterized by single‐crystal and powder XRD, IR, Raman, and solid‐state MAS ^19^F NMR spectroscopy, as well as thermogravimetric analysis, XPS and near‐edge X‐ray absorption fine structure (NEXAFS) spectroscopy.

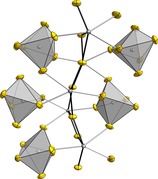

## Introduction

The binary lead fluorides PbF_2_ and PbF_4_ are well established compounds.^1^, ^2^ Their first lab synthesis dates back to the first half of the 19th century and the determination of their crystal structures to 1944 and 1962, respectively.^1^, ^2^, ^3^, ^4^ In addition, PbF_3_, which is better described as Pb_2_F_6_ containing Pb^II^ and Pb^IV^ atoms, was reported.^5^ While for Sn and Ge also the mixed valence compounds *M*_3_F_8_ (*M*=Sn, Ge), and even Ge_5_F_12_ and Ge_7_F_16_ are known, only the three binary lead fluorides mentioned above are unambiguously known.^6^, ^7^, ^8^, ^9^ Therefore, the existence of a mixed valence compound of the composition Pb_3_F_8_ appears to be likely. For lead, mixed valence compounds are nothing special and the well‐characterized compound Pb_3_O_4_ (*latin*: Minium), which was used as a pigment in ancient Rome and in anti‐corrosion coatings, or which is even today in usage for charlatanism, comes to the mind.^10^, ^11^, ^12^, ^13^ A compound of the average chemical composition {Pb_3_F_8_} was mentioned only twice in the literature. Nothing besides this average composition has been reported. Pb_3_F_8_ was first mentioned in 1972 by Banner and co‐workers as a result of the reaction of Pb_3_O_4_ with gaseous HF on a thermogravimetric scale.^14^ In their search for Pb_2_F_6_, Charpin and co‐workers described reactions leading to Pb_3_F_8_ as a product or side product. Again, no details on Pb_3_F_8_ were given, even not how the compound was identified as Pb_3_F_8_.^15^ Herein, we present the synthesis and characterization of the binary lead(II/IV) fluoride Pb_3_F_8_.

## Results and Discussion

The formation of the title compound can be envisaged by the following stoichiometric Equation (1).

(1) $$\mathrm{Pb}{}_{3}O{}_{4}+8\;\mathrm{HF}\to \mathrm{Pb}{}_{3}F{}_{8}+4\;H{}_{2}O$$

Pb_3_O_4_ is reacted with an excess of anhydrous HF (aHF) at room temperature, so that the equilibrium of the reaction is shifted to the product side. After a few minutes of reaction time the deep‐orange color of Pb_3_O_4_ is already gone and the reaction is complete within one hour at room temperature. After the removal of the volatiles (HF and H_2_O), the product is obtained as a slightly beige powder (Figure S1, Supporting Information) that is easily ground. The dry powder of Pb_3_F_8_ is stable for several hours in air. The compound prepared in this way always contains small amounts of PbF_2_ (typically 5–8 %), as evidenced by Rietveld analysis (Table S1, Figure S2, Supporting Information) on powder X‐ray diffraction patterns. The obtained lattice parameters are *a=*8.8434(1), *b=*7.5427(1), *c=*10.2339(1) Å, *β*=98.810(1)°, *V=*672.3(3) Å^3^ at *T=*298 K. They agree well with those obtained from single‐crystal X‐ray diffraction, see below. To suppress the back reaction by hydrolysis, a large excess of circa 100 equivalents of aHF is needed. If the reaction mixture is allowed to stand for three days at room temperature, or, if an excess of aHF is used that is too small, a product is obtained that always contains larger amounts of PbF_2_ than mentioned above. To obtain phase pure Pb_3_F_8_ we attempted to oxidize PbF_2_ using an excess of BrF_3_ under warming up to 130 °C. However, after evaporation of the residual BrF_3_, the remaining colorless powder consists of Pb_3_F_8_ and small amounts of Pb_2_F_6_ (circa 3 %). Thermogravimetric investigations (Figures S3, S4, Supporting Information, and for further details see the Supporting Information) indicate that the thermal decomposition of Pb_3_F_8_ is complex. The decomposition under loss of fluorine gas starts roughly around 80 °C. After thermal decomposition, pure PbF_2_ is obtained as evidenced by powder XRD (Figure S5, Supporting Information). The overall mass loss during this procedure has been determined twice, once to 5.2 and once to 4.7 %. Both values are in reasonably good agreement with the theoretically expected mass loss of 4.9 %. Thus, Pb_3_F_8_ decomposes thermally to three equivalents of PbF_2_ and one equivalent of F_2_. Further details will be reported elsewhere. Helium pycnometric density determination (see the Supporting Information) yields a density of circa 7.68 g cm^−3^ for the used sample of Pb_3_F_8_. Due to the presence of circa 15 % PbF_2_ (*ρ*=8.44 g cm^−3^) in the sample used for density determination, a value of 7.74 g cm^−3^ is to be expected from the measurements. Thus, the experimentally determined density is in very good agreement with the measurement and with the crystallographic density of Pb_3_F_8_ of circa 7.61 g cm^−3^.

**Figure 1:**
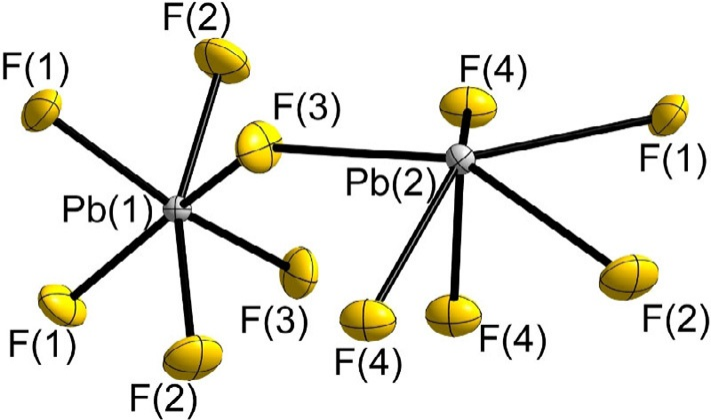
The coordination spheres of the two lead atoms of Pb_3_F_8_. The Pb(1) atom is coordinated octahedron‐like, the Pb(2) atom like a pentagonal pyramid. Pb atoms are shown in grey, F atoms in yellow. Displacement ellipsoids at 70 *%* probability at 100 K.

Single‐crystal X‐ray diffraction shows Pb_3_F_8_ to crystallize in the monoclinic space group *I*2/*a* (No. 15, *mS*44, 15*ef* ^5^) with the lattice parameters *a=*8.7800(18), *b=*7.4927(15), *c=*10.196(5) Å; *β*=98.78(3)°; *V=*662.9(4) Å^3^; *Z=*4, at *T=*100 K, while at room temperature lattice parameters of *a=*8.8400(5), *b=*7.5398(5), *c=*10.2297(7) Å, *β*=98.82(2)°, *V=*673.77(8) Å^3^ are obtained. The latter agree well with the values determined from powder X‐ray diffraction at room temperature. No phase change was observed upon cooling from room temperature to 100 K and Table S2, Supporting Information, holds details of the single crystal structure determination. Surprisingly, Pb_3_F_8_ is not isotypic to the compounds *M*_3_F_8_ (*M*=Ge, Sn) but, to the best of our knowledge, represents a novel structure type.^6^, ^7^ As the crystal structure of Pb_3_F_8_ is complicated we will start with the local structure description before we describe it globally. There are two types of Pb atoms, Pb(1) and Pb(2). The Pb(1) atoms (Wyckoff position 4*e*) are coordinated by F atoms (8*f*) in the shape of an irregular octahedron, while the coordination polyhedron around the Pb(2) atom (8*f*) reminds of a pentagonal pyramid (Figure 1).

All F atoms around the Pb(1) atom are μ_2_‐bridging to Pb(2) atoms. The Pb(1)–F atomic distances are observed in the range from 2.048(3) to 2.063(3) Å. They agree well with reported ones for hexafluoridoplumbates(IV) in compounds such as *M* ^II^PbF_6_ (*M* ^II^=Mg (1.99 Å), Ni (1.99 Å), Zn (1.97 Å), Sr (2.042–2.060 Å), Ba (2.04 Å), Pb (1.991–2.011 Å)) or *M* ^I^_2_PbF_6_ (*M* ^I^=Ag (2.021–2.100 Å), Li (1.997 Å)), which however all contain spatially separated [PbF_6_]^2−^ octahedra.^16^, ^17^, ^18^, ^19^, ^20^ Therefore, we assign oxidation state +IV to these octahedron‐like coordinated Pb(1) atoms. As stated above, the Pb(2) atoms are coordinated by six fluorine atoms in a shape similar to a pentagonal pyramid (Figure 1) and the Pb(2)–F distances span a rather broad range from 2.330(3) to 2.651(3) Å. As they are clearly longer than the Pb(1)–F distances, we assign oxidation state +II to the Pb(2) atoms. Charge distribution (CHARDI) calculations^21^ (Table S3, Supporting Information) agree with the description of Pb_3_F_8_ as a mixed valence compound as charges of +4.12 and +1.94 are calculated for the Pb(1) and Pb(2) atoms, respectively. Thus, the assignment of the oxidation states is supported.

One Pb(2)–F(4) distance within the pentagonal pyramid is shortest with 2.330(3) Å, and represents the “tip” of the pyramid pointing to the bottom in Figure 1. The other two Pb(2)–F(4) distances are longer and equal within the standard uncertainty (2.446(3) and 2.449(3) Å). The other Pb(2)–F distances are significantly longer and range from 2.505(3) to 2.651(3) Å. As can be seen in Figure 1, the Pb(2) atom is not located in the center of the coordination polyhedron but resides close to the pentagonal face. Such a coordination polyhedron is reminiscent of the text‐book anion [XeOF_5_]^−^,^22^, ^23^, ^24^ and the peculiar location and coordination sphere of the Pb(2) atom is attributed to an accumulation of electron density in real space as shown in the quantum chemical calculations below. Due to the chemical hardness of the fluoride anion and its extremely low polarizability, its electron density leads to repulsion and deformation of the electron density at the Pb atom. Some call this effect the “sterically active lone‐pair” and its influence on local as well as crystal structure is known for example from α‐ and β‐PbO, or from the black and pigeon blood red modifications of SnO.^25^, ^26^, ^27^ However, above the “lone‐pair” of the Pb^II^ atom there are three additional F atoms with Pb–F distances of 2.851(4), 2.874(3), and 3.051(3) Å. According to the distance histogram one could count those three F atoms to the coordination sphere of Pb(2) leading to coordination number 6+3. The coordination polyhedron around Pb(2) is then irregular with ten triangles and one tetragon as the faces. Also, the calculated effective coordination number (ECoN) of 6.9 hints to a small contribution of the three next‐nearest fluorine atoms to its coordination sphere, whereas the calculated ECoN for Pb(1) agrees well with C. N.=6 as assigned by our structure analysis.

**Figure 2:**
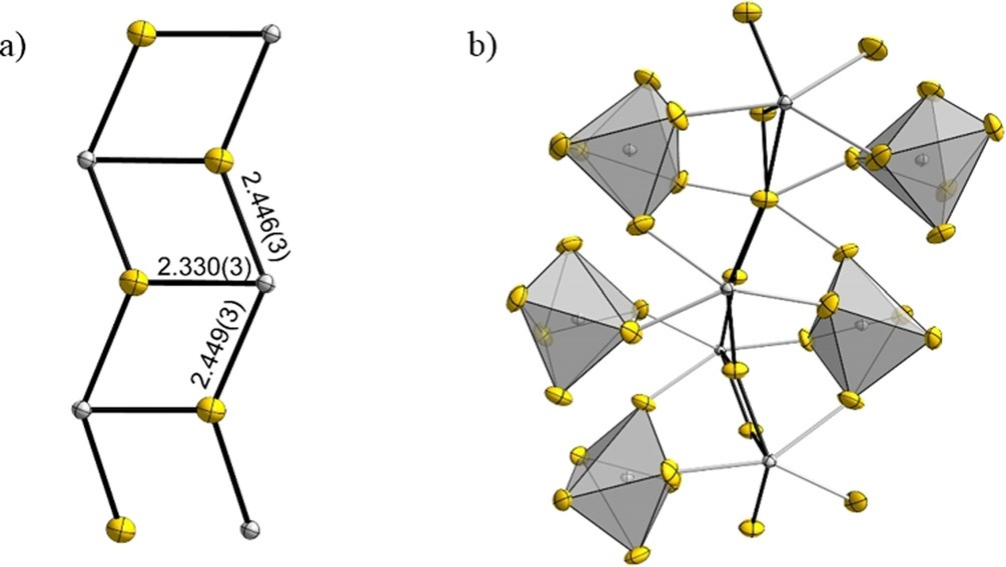
a) Ladder‐like connection of the Pb(2) atoms (grey) via μ_3_‐bridging F(4) atoms (yellow). b) Connection of the Pb(2) containing ladder to the Pb(1) containing octahedra. Displacement ellipsoids shown with 70 *%* probability at 100 K.

We will now come to the global structure description by explaining how the coordination polyhedra are interconnected. The F(4) atoms are μ_3_‐bridging between Pb(2) atoms and that leads to the formation of a 1D infinite zigzag ladder shown in Figure 2 a. The two longer Pb(2)–F(4) distances form the stringers of the ladder, while the short Pb(2)–F(4) distances represent the rungs of the ladder (Figure 2 a). Thus, the “lone‐pairs” on the Pb(2) atoms point to the left and right in Figure 2 a.

**Figure 3:**
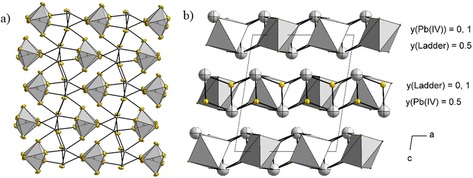
a) A part of the 2D infinite layer formed by the sandwiching of [Pb(1)F_6_]^2*−*^ octahedra by Pb(2) containing ladders. Displacement ellipsoids at 70 *%* probability at 100 K. b) A section of the crystal structure of Pb_3_F_8_. Atoms are shown isotropic with arbitrary radii. Pb atoms grey, F atoms yellow. The 2D infinite layers run parallel to the ab plane. The height along the b axis is shown with the approximate *y* coordinate of the gravimetric center of the building units.

The topside and underside of the infinite ladder are coordinated by [Pb(1)F_6_]^2−^ octahedra as shown in Figure 2 b. The ladders are sandwiched between the octahedra and vice versa, leading to a 2D infinite layer of ladders interconnected by octahedra. A section is shown in Figure 3 a.

Thus, the “sterically active lone‐pairs” of the Pb(2) atoms point out of the topside and underside of these layers (Figure 3 b) separating them from each other. Figure 3 b shows a section of the crystal structure of Pb_3_F_8_ with the 2D infinite layers parallel to the *ab* plane stacked along the *c* axis. The *Niggli* formula indicates the coordination number and environment of the Pb atoms nicely. For the Pb^II^ atom [PbF_3/2_F_3/3_] and for the Pb^IV^ atom [PbF_6/2_] is obtained. Thus, Pb_3_F_8_ can be described by the *Niggli* formula 2∞ [PbF_3/2_F_3/3_]_2_[PbF_6/2_]. The Pb atoms are hexagonally packed and each is anticuboctahedrally surrounded by twelve Pb atoms. Thus, the arrangement of the Pb atoms of Pb_3_F_8_ corresponds to the simple Mg structure type. However, the F atoms neither fill the octahedral nor the tetrahedral voids of the sphere packing.

Raman spectroscopic investigations have been carried out on Pb_3_F_8_ and on PbF_2_ for comparison. For experimental details see the Supporting Information. The experimentally obtained spectra were then compared with ones obtained from DFT‐PBE0/TZVP calculations based on the crystal structures of Pb_3_F_8_ and PbF_2_. The most striking difference between the Raman spectrum of Pb_3_F_8_ and the spectrum of PbF_2_ (see Figures S6 and S7, Supporting Information) is the strong vibrational band at 531 cm^−1^ that is only present in the Raman spectrum of Pb_3_F_8_. This band is well reproduced by our theoretical findings and can be attributed to a symmetric stretching of the Pb^IV^−F bonds, which explains the absence of this band in PbF_2_. Pb_3_F_8_ is also clearly identified by the lattice vibrational bands around 100 cm^−1^ as this frequency region corresponds to a minimum in Raman intensity in the spectrum of PbF_2_. The two peaks at around 250 cm^−1^ and the two peaks at around 200 cm^−1^ belong to a symmetric stretching of the Pb^II^−F bonds and bending modes of the Pb^IV^−F bonds, respectively. In summary, the Raman spectrum supports our classification of Pb_3_F_8_ as a mixed valence compound. Full band assignments are available from Tables S4 to S6, Supporting Information.

An IR spectroscopic investigation of Pb_3_F_8_ powder in the range from 4000 to 450 cm^−1^ (Figure S8) shows only a single broader band at 466 cm^−1^, which is comprised of intense Pb^IV^–F stretch and weaker Pb^II^–F scissoring and rocking modes. For Li_2_PbF_6_, which contains [PbF_6_]^2−^ octahedra, a band at 475 cm^−1^ has been observed.^16^ This agrees well considering the different connectivity of the [PbF_6_]^2−^ octahedra in the two compounds. The experimentally determined band position of Pb_3_F_8_ agrees well with the quantum chemically calculated bands at 493, 470, and 456 cm^−1^. The complete assignment of IR bands is given in Table S5, Supporting Information. The obtained Pb_3_F_8_ is essentially free of impurities such as H_2_O, OH^−^, or HF, as no bands in the range from 4000 to circa 450 cm^−1^ are present.

**Figure 4:**
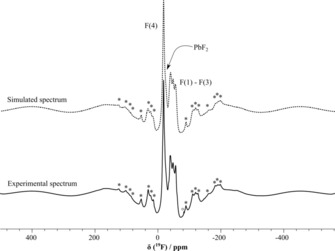
^19^F DEPTH MAS NMR spectrum (experimental: solid line, simulated: dashed line) of Pb_3_F_8_ at 20 kHz spinning frequency. The spinning side bands are marked with asterisks. The simulation includes a version of the DEPTH^[32, 33]^ sequence with four π‐pulses: π/2*‐*π*‐*π*‐*π*‐*π‐τ_deadtime_‐FID. The DEPTH experiment results in MAS NMR spectra free of probe head background. The simulation includes the effect of the deadtime delay and excitation profile of the DEPTH sequences which causes the baseline rolling. Zeroth and first order phase correction are included as variable parameters in the least‐square fit.

Solid‐state ^19^F MAS NMR experiments (Figure 4 and Table 1) of Pb_3_F_8_ were performed to further corroborate the crystal structure model. The ^19^F DEPTH MAS NMR spectrum shows four resonances, one occurring at *δ*=−18.2 ppm and a group of three overlapping signals at *δ*=−40, −48.5, and −56 ppm. All four resonances have peak areas including spinning sidebands of 1:0.84:1.03:0.90. The spectrum also contains a fifth peak at *δ*=−24.2 ppm with a lower intensity which is likely to originate from the PbF_2_ impurity.^28^ These observations are expected for F atoms which do not have fast ion‐dynamics on the NMR timescale, as four symmetry‐inequivalent F atoms (F(1) to F(4)) with the same site multiplicity are present in the crystal structure.

**Table 1:** Estimates for the ^19^F solid‐state NMR chemical shift parameters for Pb_3_F_8_ obtained by a least‐square fit of the experimentally obtained spectrum (Figure 4) with SIMPSON version 3.1.2^29^ simulations of the used version of the DEPTH^30^, ^31^ experiment.

Site	*δ*_iso_ [ppm]	*δ*_aniso_ [ppm]	*η*	*δ*_11_ [ppm]	*δ*_22_ [ppm]	*δ*_33_ [ppm]
F(4)	−18.2	66.1	0.60	47.9	−31.5	−71.2
F(1)–F(3)	−40.0	−111.8	0.61	50.5	−18.5	−151.7
F(1)–F(3)	−48.5	−121.0	0.65	51.5	−27.4	−169.5
F(1)–F(3)	−56.0	−119.2	0.47	31.6	−24.5	−175.2

A tentative peak assignment of the ^19^F resonances follows the idea that neighboring cations contribute to the ^19^F chemical shift according to their coordination number and distance to F atoms in ionic fluorides.^32^ Consequently, F atoms with a similar bonding situation should feature similar isotropic and anisotropic chemical shift values. In the present case (Table 1) the group of three resonances has an anisotropic chemical shift which is larger by about a factor of two compared to the peak which appears at the highest ppm values. In the structure three F atoms are coordinated to two Pb atoms, one F atom is coordinated to three. Therefore, the resonance at −18.2 ppm is assigned to the three‐fold coordinated fluorine site (F(4)) and the three signals at −40, −48.5, and −56 ppm are assigned to the fluorine atoms F(1), F(2), and F(3) coordinated by the two lead Pb(1) and Pb(2) atoms.

**Figure 5:**
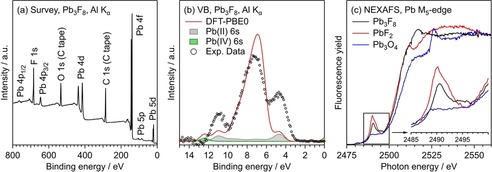
(a) Survey XP spectrum of Pb_3_F_8_ on carbon tape, taken with monochromatic Al K_*α*_ radiation. (b) Valence band spectrum of Pb_3_F_8_ measured with Al K_α_ radiation in comparison to DFT calculations (PBE0/NCPW). A Shirley background was subtracted from the experimental data to compare it to the theoretical results. The contribution of the Pb 6s orbitals to the total calculated DOS is highlighted. Further details concerning the data treatment are given in the Supporting Information. (c) Pb M_5_‐edge NEXAFS spectra of Pb_3_F_8_, PbF_2_ and Pb_3_O_4_ measured by the X‐ray fluorescence yield. Inset: Zoom‐in of the pre‐edge feature.

We have performed X‐ray photoelectron spectroscopy (XPS) as well as near‐edge X‐ray absorption fine structure (NEXAFS) measurements to get information about the electronic structure of Pb_3_F_8_. The survey XP spectrum of Pb_3_F_8_ on carbon tape is presented in Figure 5 a. The spectrum only shows contributions from Pb and F atoms, besides minor C 1s and O 1s peaks from the carbon tape.

Another sample that was studied with hard X‐ray photoelectron spectroscopy (HAXPES, Figure S9 in the Supporting Information) shows the same features and even less contributions from the carbon tape. During the XPS and HAXPES measurements, the sample exhibits substantial photoemission‐induced charging, which results in peak shifts and broadening. For this reason, a refined analysis of the XPS peak shapes with a discrimination between Pb^II^ and Pb^IV^ contributions is not possible. Instead, we performed NEXAFS spectroscopy measurements on the Pb M_5_‐edge to gain further insight into the electronic structure of Pb_3_F_8_ (Figure 5 c). As a reference, we also studied PbF_2_ and Pb_3_O_4_. Between 2490 and 2495 eV, a pre‐edge feature is observed, which is followed by the M_5_‐edge for all three species. PbF_2_ shows a sharp peak at 2490 eV with a minimum at 2495 eV. In contrast, there is only a broad feature between 2490 and 2495 eV for Pb_3_O_4_. The Pb_3_F_8_ spectrum resembles a mixture of both reference samples. A peak at 2490 eV is observed, whereas there is no minimum at 2495 eV like for PbF_2_. Instead, there is a broad feature similar to the case of Pb_3_O_4_. This is in line with the presence of both Pb^II^ and Pb^IV^ species in the Pb_3_F_8_ sample and with a small contamination of PbF_2_, as stated above. The differences in the M_5_‐edge itself are more complicated as there are nearly no similarities between the three compounds. In the range from 2500 to 2510 eV, PbF_2_ and Pb_3_F_8_ show similar spectral features, but above that range PbF_2_ exhibits a local minimum, whereas Pb_3_F_8_ shows a peak. A similar peak is observed in the Pb_3_O_4_ spectrum but shifted by nearly 10 eV to higher energies.

We have calculated the electronic structure of Pb_3_F_8_ by DFT methods using the hybrid functional PBE0 and fully relativistic pseudopotentials.^33^, ^34^ To estimate the accuracy of our calculations we compared the experimentally determined valence band XP spectrum with the calculated partial density of states (pDOS) that is corrected by background and cross‐section effects (see the Supporting Information). The results are shown in Figure 5 b. The valence band width as well as its three‐peaked shape are well reproduced by the DFT calculations.

**Figure 6:**
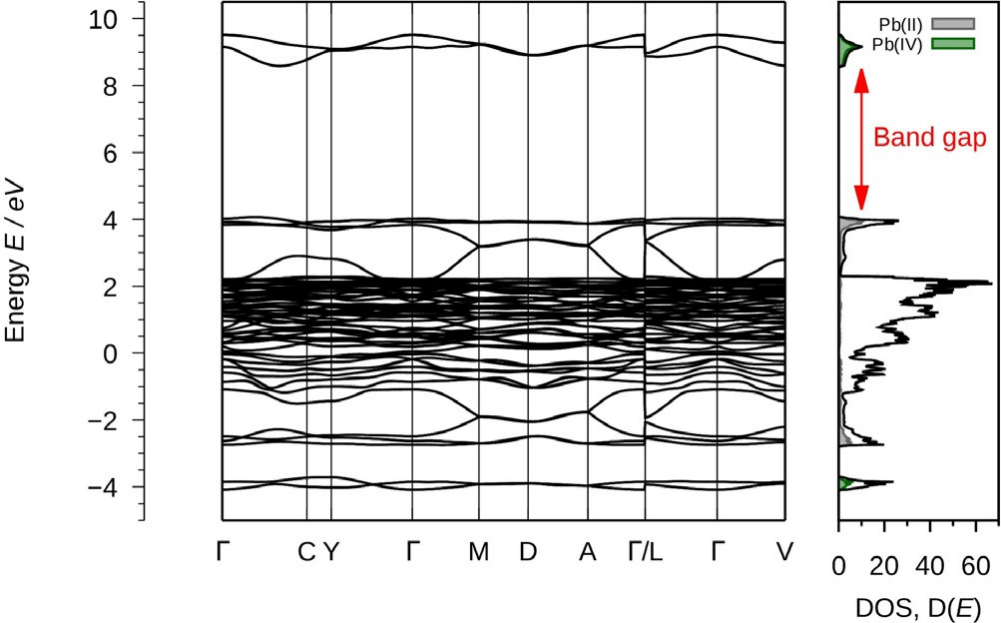
Left: Electronic band structure of Pb_3_F_8_. Right: Total Density of States (DOS) and the projected DOS of the 6s orbitals of Pb^II^ and Pb^IV^. The position of the band gap (4.5 eV) is highlighted (DFT‐PBE0/NCPP with SOC).

In the following, we investigate the electronic structure of Pb_3_F_8_ in more detail by calculating its band structure and charge distribution. The band structure as well as the total DOS are given in Figure 6.

The band structure calculations show Pb_3_F_8_ to be an insulator with a band gap of approximately 4.5 eV in line with its off‐white color. The DOS of the valence band is dominated by F 2p states that range from −1 eV to 2 eV. At about 4 eV the top of the valence band consists of four bands with only a small amount of dispersion that can be attributed to the filled Pb^II^ 6s bands. The conduction band is located at about 9 eV and consists of two bands. Both show nearly exclusive Pb^IV^ 6s character as illustrated by the pDOS in the right of Figure 6. A small amount of the Pb^IV^ 6s states is located at the bottom of the valence band at about −4 eV due to some covalent Pb^IV^−F bond character. For the same reason Pb^II^ 6s states are present at about −2 eV. The band structure of the mixed valence compound Pb_3_O_4_ shows similar characteristics.^11^ We thus conclude that like Pb_3_O_4_ also Pb_3_F_8_ is a mixed valence compound with the lead atoms in the oxidation states +II and +IV.

**Figure 7:**
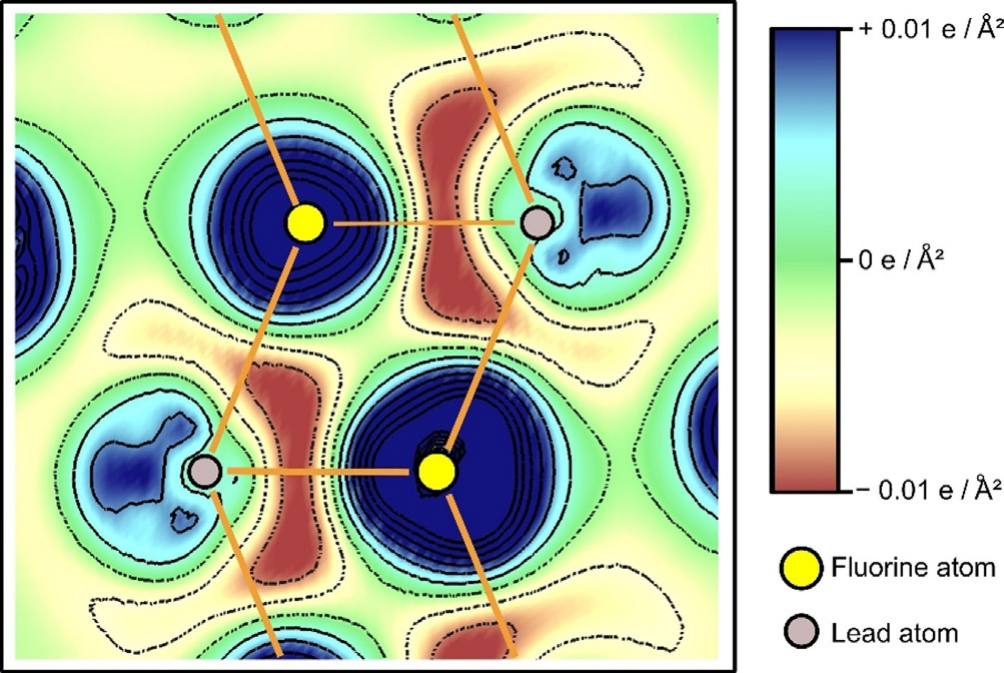
Electron‐density difference map (DFT‐PBE0/NCPP+SOC) of Pb_3_F_8_ along the ladder‐like connection (sketched) of the Pb(2) atoms (grey color) via *μ*_3_‐bridging F(4) atoms (yellow color). An increase in electron density is shown in blue color and solid black lines, while a decrease in electron density is shown in brown color and dashed black lines.

The crystal structure of Pb_3_F_8_ indicates that the Pb^II^ atoms feature “sterically active lone‐pairs”. We calculated electron‐density difference maps of Pb_3_F_8_ which display the difference of the electron density of the compound compared to a superposition of the electron density of free atoms, yielding information where electron density is accumulated or depleted. The electron‐density difference map of Pb_3_F_8_ is shown in Figure 7. It is drawn in a view perpendicular to the ladder‐like connection of the Pb(2) atoms and the F(4) atoms, compare Figure 2 a.

The map displays a strong polarization of the electron density around the Pb^II^ atoms. The electron density along the Pb–F bonds is minimized (brownish colors) in line with the expected high amount of ionic bonding character. Moreover, there is an accumulation of electron density (in blue colors) besides the Pb^II^ atoms, pointing to the left and right side of the depicted ladder. Therefore, the electron density at the Pb^II^ atoms is “pushed” away from the fluorine atoms inside the ladder. This effect is often referred to “sterically active lone‐pairs” of the Pb^II^ atoms. The electron density around the fluorine atoms (in yellow color) is strongly and nearly spherically increased as is expected for F^−^ anions due to the high electronegativity of the F atom.

## Conclusions

The binary lead(II/IV) fluoride Pb_3_F_8_ was synthesized from Pb_3_O_4_ in anhydrous HF at room temperature. The bulk phase appears off‐white while single crystals are colorless. It is thermally stable up to circa 80 °C and then decomposes to PbF_2_ under loss of F_2_. The compound crystallizes in the monoclinic space group *I*2/*a* (No. 15) with the lattice parameters *a=*8.7800(18), *b=*7.4927(15), *c=*10.196(5) Å; *β*=98.78(3)°; *V=*662.9(4) Å^3^; *Z=*4 at *T*=100 K, as evidenced by single‐crystal X‐ray analysis. The description of Pb_3_F_8_ as a mixed valence Pb^II^/Pb^IV^ compound is evidenced by the thermal decomposition products, the crystal structure, the ^19^F solid‐state NMR, valence and core level photoelectron, as well as near‐edge X‐ray absorption fine structure (NEXAFS) spectroscopic investigations and further supported by IR and Raman spectra. Additionally, quantum chemical calculations were carried out to elucidate the electronic structure of Pb_3_F_8_. The calculated band gap is in line with the color of the compound. An accumulation of electron density next to the Pb^II^ atoms that some call “sterically active lone‐pairs” seems to be responsible for the formation of the peculiar layer structure of Pb_3_F_8_.

## Conflict of interest

The authors declare no conflict of interest.

## Supporting information

As a service to our authors and readers, this journal provides supporting information supplied by the authors. Such materials are peer reviewed and may be re‐organized for online delivery, but are not copy‐edited or typeset. Technical support issues arising from supporting information (other than missing files) should be addressed to the authors.

Supplementary
