# Supplementary material for: Binary Lead Fluoride Pb3F8
Source: Chemistry. 2019 Nov 4;25(68):15656–61. doi: 10.1002/chem.201903954 (PMC6916602; doi:10.1002/chem.201903954)
Supplement: Supplementary file 1 — Supplementary [file CHEM-25-15656-s001.pdf]

CHEMISTRY
A European Journal

Supporting Information

Binary Lead Fluoride Pb_3F_8

H. Lars Deubner+,[a] Malte Sachs+,[a] Jascha Bandemehr,[a] Sergei I. Ivlev,[a] Antti J. Karttunen,[b] Stefan R. Kachel,[a] Benedikt P. Klein,[a] Lukas Ruppenthal,[a] Maik Schöniger,[a] Claudio K. Krug,[a] Jan Herritsch,[a] J. Michael Gottfried,[a] Jamal N. M. Aman,[c] Jörn Schmedt auf der Günne,[c] and Florian Kraus*[a]

chem_201903954_sm_miscellaneous_information.pdf

Contents

Experimental Procedures 2
Synthesis of Pb_3F_8 2
Powder X-Ray Diffraction 2
Density determination 4
Single crystal X-ray diffraction 5
Thermal analysis 6
CHARDI Calculations 8
Raman Spectroscopy 9
IR spectroscopy 13
Solid-state NMR spectroscopy 14
XPS, HAXPES and NEXAFS 15
Quantum chemical calculations 17
Basis set details for the CRYSTAL17 calculations 19
Structural Optimization 20
Optimized geometries in CRYSTAL input format 22
Literature 23

Experimental Procedures

General: All operations were carried out in an atmosphere of dry and purified argon (5.0 Praxair, Germany), so that possible contact of the substances with moisture and air was minimized. Anhydrous HF was additionally dried by mixing it with K_2NiF_6, which reacts with traces of moisture, and separated by vacuum distillation in a Monel Schlenk line. Pb_3O_4 (Merck, ≥ 99 %) was used without further purification.

Synthesis of Pb_3F_8

A FEP tube with a stainless-steel valve was charged with Pb_3O_4 (250 mg, 0.365 mmol). A large excess of anhydrous HF (ca. 100 eq. compared to Pb_3O_4) was condensed on the Pb_3O_4 powder at −196 °C. The reaction vessel was slowly warmed to room temperature and kept for 1 h under sporadic shaking after the orange color had disappeared. Then, the volatiles (HF and H_2O) were carefully removed *in vacuo* at room temperature. The isolated product, which was used for further characterization, weighed 281 mg (0.363 mmol, 99.4 % of theory). Some small single crystals were always obtained among the powder.

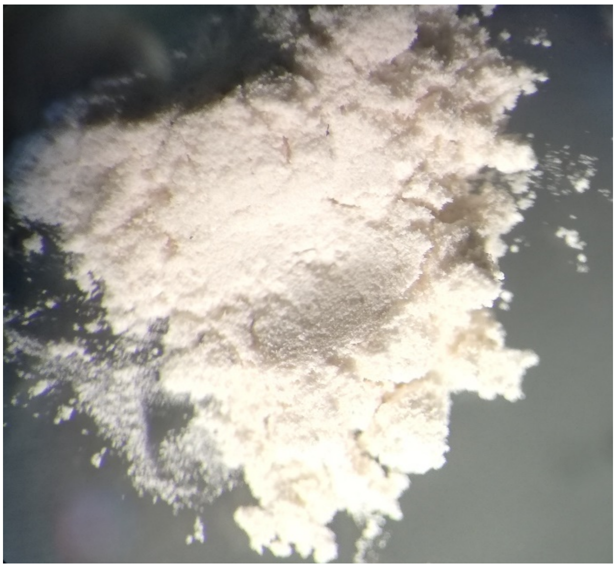

Figure S 1. Pb_3F_8 powder after removal of the volatiles (HF and H_2O).

Powder X-Ray Diffraction

The powder X-ray pattern was recorded with a StadiMP diffractometer (Stoe & Cie) in transmission geometry using a flat foil sample holder. The diffractometer was operated with Cu $K_{\alpha1}$ radiation (1.5406 Å, germanium monochromator) and equipped with a MYTHEN 1K detector. The diffraction pattern was indexed using the WinXPOW suite.[1] Rietveld refinement was done with Topas-Academic V6.[2] The powder diffraction pattern with the Rietveld refinement is shown in Figure S 2.

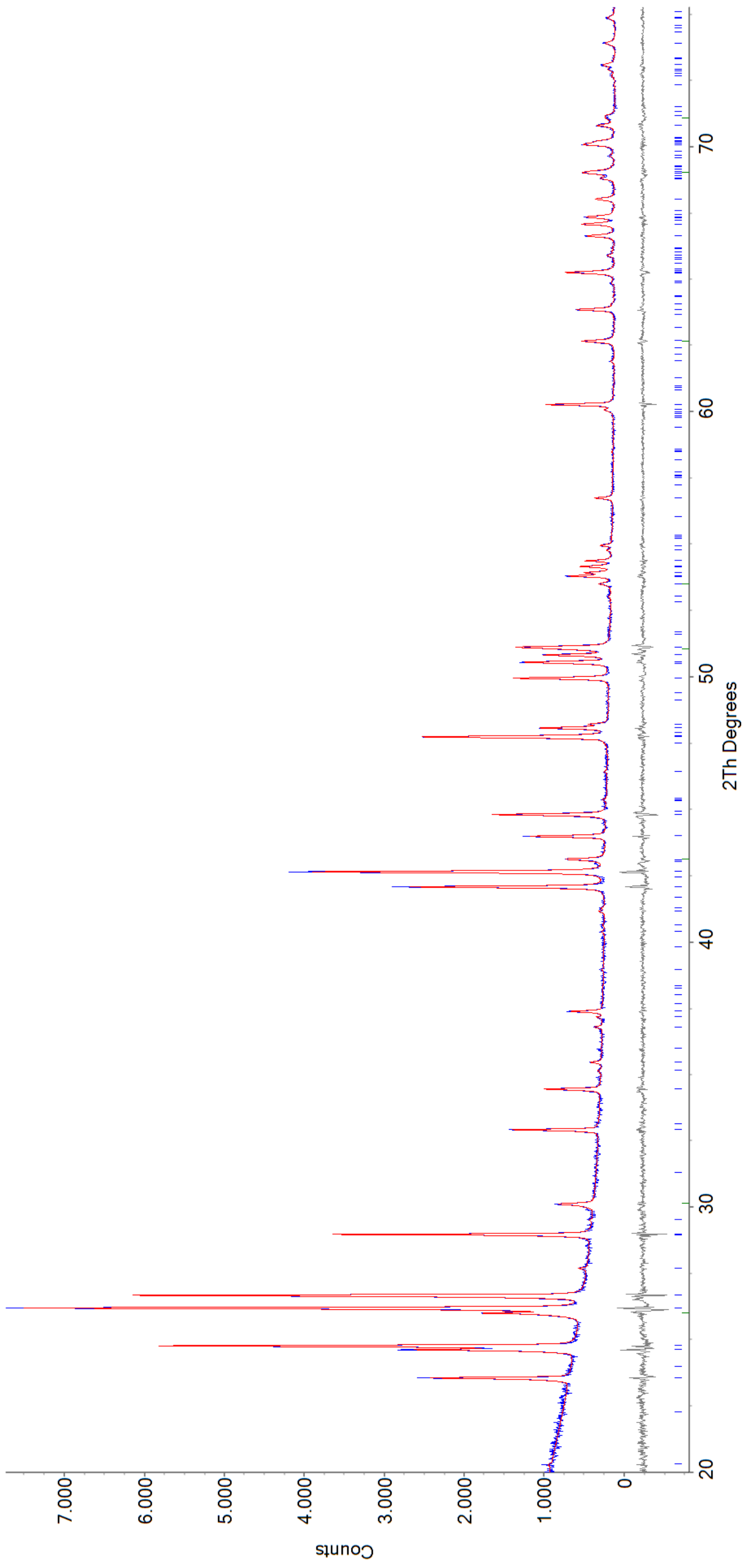

Figure S 2. Powder diffraction pattern of a Pb_3F_8 flat sample. The measured powder pattern is shown in blue, the fitted pattern (Rietveld refinement) is shown in red, the grey curve on the bottom shows the difference curve. The refined lattice parameters for Pb_3F_8 are a = 8.84344(8), b = 7.54266(8), c = 10.23393(11) Å, β = 98.8124(6)°, V = 674.580(12) Å^3. GOF 1.187, R_p = 0.0467, R_{wp} = 0.0611.

Table S 1. Technical data of the Rietveld refinement of Pb_3F_8.

Measurement conditions	
Sample	Pb_3F_8
Diffractometer, generator	Stoe STADI MP; 40 kV, 40 mA
Diffractometer geometry	Transmission (flat foil)
Radiation, monochromator	Cu $K_{\alpha 1}$, Ge(111)
Measurement range, step size (2θ / °)	20−75, 1.00
Measurement time per step / s	15.0
Data acquisition	step scan
Temperature *T* / K	298
Global parameters	
Software	TOPAS-Academic v6
Number of phases	2
Relative phase amounts in mass / %	Pb_3F_8 93.49(8), PbF_2 6.51(8)
Number of parameters	58
Zero shift (2θ / °)	0 (within 3σ)
Profile function	TCHZ Pseudo-Voigt
Asymmetry	TOPAS Simple axial model
Background treatment	Chebyshev polynomial of 9th order
Structural data	
Space group (No.)	*I*2/*a* (15)
Pearson symbol	*mS*44
a / Å	8.84344(8)
b / Å	7.54266(8)
c / Å	10.23393(11)
β / °	98.8124(6)
V / Å^3	674.580(12)
Crystallographic density / g cm^{-1}	7.61707(13)
Profile parameters	
Peak.shape parameter *V*	0.05689(16)
Peak.shape parameter *W*	0.05931(19)
Peak.shape parameter *Z*	0.070(2)
Peak.shape parameter *X*	0.1024(12)
Asymmetry parameter	8.86(6)
Global residual values	
R_p	0.0467
R_{wp}	0.0611
R_{Bragg}	0.0137
GOF	1.186

Density determination

The density of Pb_3F_8 was measured using the automated gas displacement pycnometry system AccuPyc II 1340 (Micromeritics) with a calibrated 1 cm^3 sample holder and helium as the displacement gas. The number of preliminary purges was set to 30, while the subsequent density measurements were performed 100 times with measurement averaging. The density of the sample was determined to be 7.682(16) g·cm^{-3}, the sample contained 84.5(9) % Pb_3F_8 and 15.5(9) % PbF_2. A total sample mass of 855.6 mg was used.

Single crystal X-ray diffraction

A crystal of Pb_3F_8 was selected under pre-dried perfluorinated oil and mounted using a MiTeGen loop. Intensity data of a suitable crystal were recorded with an IPDS 2T diffractometer (Stoe & Cie) at an offset of 30°. The diffractometer was operated with Mo K_α radiation (0.71073 Å, graphite monochromator) and equipped with an image plate detector. Evaluation, integration and reduction of the diffraction data was carried out using the X-Area software suite.[3] A numerical absorption correction was applied with the modules X-Shape and X-Red32 of the X-Area software suite. The structure was solved with dual-space methods (SHELXT-2014/5) and refined against F^2 (SHELXL-2014/7).[4,5] All atoms were refined with anisotropic displacement parameters. The highest residual electron density of 1.380 e·Å^{-3} after the final refinement was 1.2 Å distant from atom Pb(1). The cif file was deposited with the CCDC (https://www.ccdc.cam.ac.uk/), depository number: 1945512.

Table S 2. Selected crystallographic data and details of the structure determination of Pb_3F_8.

	Pb_3F_8
Colour and appearance	colorless
Molecular mass /g·mol^{-1}	773.56
Crystal system	monoclinic
Space group (No.)	*I*2/*a* (15)
Pearson code	*mS*44
Wyckoff sequence	15*ef*^{5}
a /Å	8.7800(18)
b /Å	7.4927(15)
c /Å	10.196(5)
α /°	90
β /°	98.78(3)
γ /°	90
V /Å^3	662.9(4)
Z	4
λ /Å	0.71073
T /K	100(2)
μ(Mo K_α) /mm^{-1}	76.100
R_{int}, R_σ	0.0594, 0.0252
$R(F)$ ($I \geq 2\sigma(I)$, all)	0.0195, 0.0279
$wR(F^2)$ ($I \geq 2\sigma(I)$, all)	0.0358, 0.0382
S (all data)	1.133
Data, parameters, restrains, constraints	1021, 52, 0, 0
$\Delta\rho_{max}$, $\Delta\rho_{min}$ /e·Å^{-3}	1.380, −1.599

Thermal analysis

Thermal measurements were performed with a DSC-TGA 3 (Mettler Toledo) with a heating rate of 1 and 0.1 K·min^{-1} in a stream of nitrogen (20 sccm). The diagram was plotted with OriginPro 2017.[6]

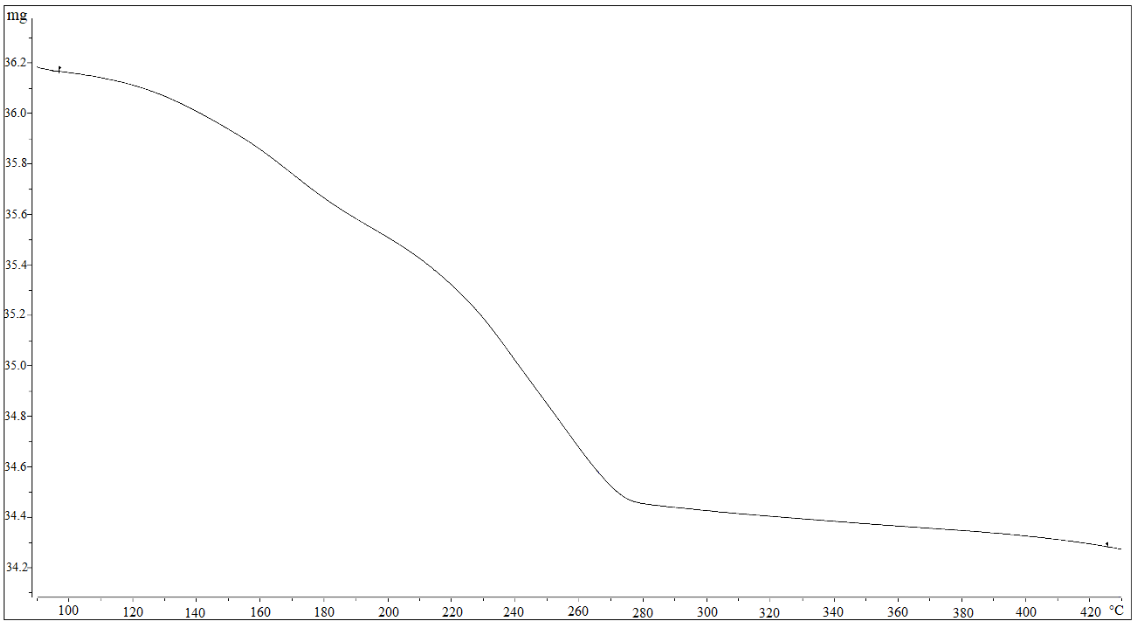

Figure S 3. Thermogravimetric measurement from 80 to 430 °C under a stream of nitrogen with a heating rate of 0.1 K·min^{-1}.

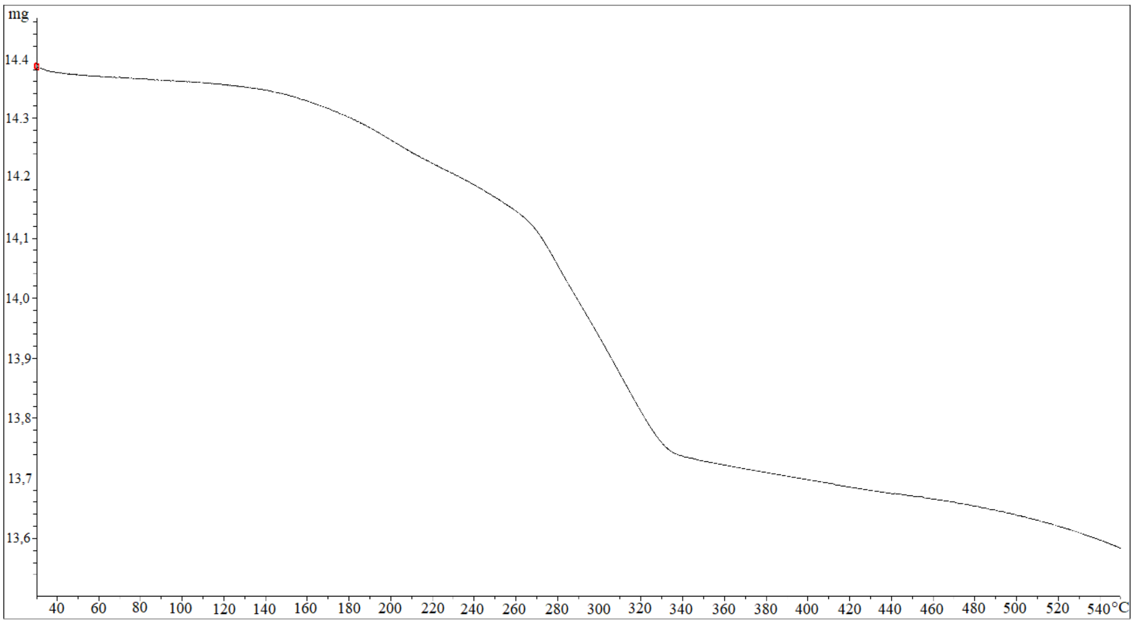

Figure S 4. Thermogravimetric measurement from 30 to 550 °C under a stream of nitrogen with a heating rate of 1 K·min^{-1}.

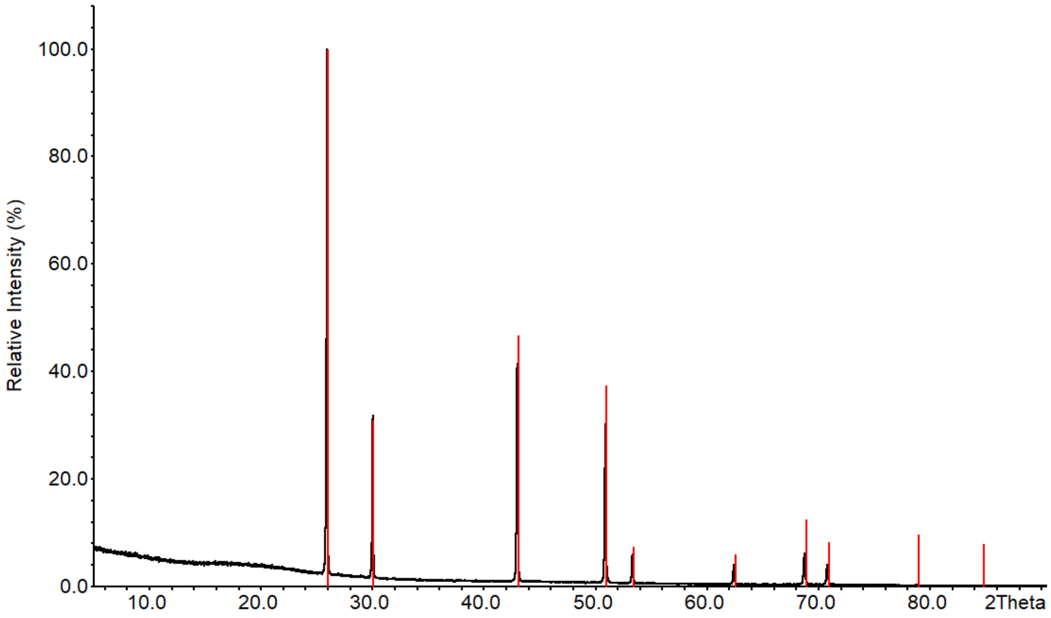

Figure S 5. Powder X-ray diffraction pattern of the product of the thermal decomposition of Pb_3F_8 powder after heating to 470 °C for 12h and subsequent cooling to room temperature. The sample was flame sealed under vacuum into a borosilicate glass ampoule which was heated inside a tube furnace.

CHARDI Calculations

We applied the charge distribution method (CHARDI) introduced by Hoppe and coworkers as implemented in the program CHARDI2015[7] to validate the assignment of the coordination numbers as well as the oxidation states of the lead atoms. We used the crystal structure data determined in this paper as input parameters. The results are summarized in Table S 3.

Table S 3. Results of CHARDI calculations of Pb_3F_8 (*I*2/*a*, *mS*44). Coordination number (C.N.) as discussed above, effective coordination number (ECoN), mean fictive ionic radius (MEFIR), assigned oxidation state based on structure-chemical reasoning, and calculated charge distribution (CHARDI).

Atom	C.N.	ECoN	MEFIR / Å	Oxidation state	CHARDI
Pb(1)	6	6.0	0.886	+IV	+4.12
Pb(2)	6 [+3]	6.9	1.344	+II	+1.94
F(1)	2	1.2	1.209	−I	−1.01
F(2)	2	1.1	1.211	−I	−1.10
F(3)	2	1.4	1.191	−I	−0.97
F(4)	3	2.9	1.113	−I	−0.93

The CHARDI calculation supports the description of Pb_3F_8 as a mixed valence compound. The calculated mean fictive ionic radii (MEFIR) of Pb(1) (0.886 Å) and Pb(2) (1.344 Å) are in line with the Shannon ionic radii of Pb(IV) (0.775 Å) and Pb(II) (1.19 Å) for coordination number six. Therefore, the CHARDI calculations demonstrate that a valid, valence-balanced description of Pb_3F_8 is possible stating Pb_3F_8 as a mixed valence compound.

Raman Spectroscopy

We have performed Raman spectroscopy on Pb_3F_8 and PbF_2. Raman spectra were recorded with a Confocal Raman Microscope S+I MonoVista CRS+, using the 532 nm excitation line of an integrated diode laser (resolution < 1 cm^{-1}; range 50 to 9000 cm^{-1}). A sample of Pb_3F_8 was sealed inside a 0.3 mm borosilicate glass capillary, which was several times flame dried under vacuum before use.

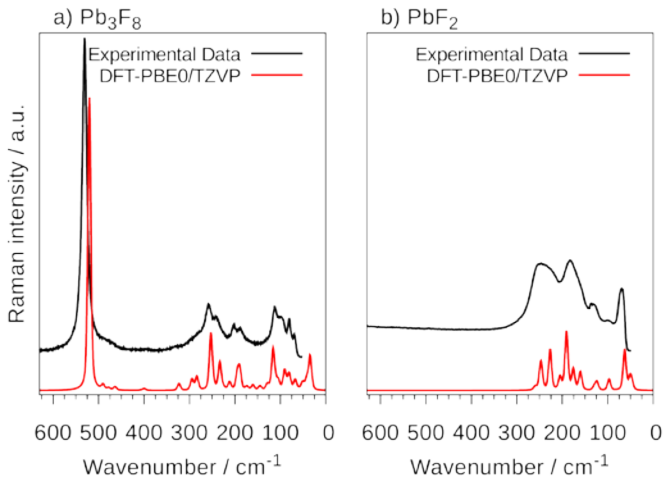

Figure S 6. **a)** Raman spectrum of Pb_3F_8 in comparison with **b)** the Raman spectrum of PbF_2. The calculated Raman intensities are shown in red (DFT-PBE0/TZVP).

The experimental results are compared with theoretical spectra from DFT calculations in Figure S 6. The observed peaks from the measured spectra are listed in Table S 4. The peak assignment of the calculated spectra of Pb_3F_8 and PbF_2 are given in Table S 5 and Table S 6, respectively. The most striking difference between the Raman spectrum of Pb_3F_8 compared to the spectrum of PbF_2 is the strong vibrational band at 531 cm^{-1} that is only present in the Raman spectrum of Pb_3F_8. This band is well reproduced by our theoretical findings and can be attributed to a symmetric stretching of the Pb(IV)−F bonds, which explains the absence of this band in PbF_2. The Raman spectrum of Pb_3F_8 thus supports our findings of Pb_3F_8 being a mixed valence compound.

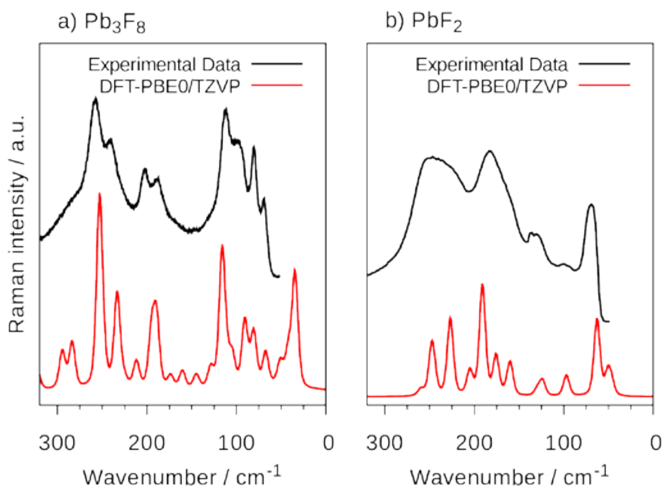

Figure S 7. Low wavenumber section of the Raman spectrum of Pb_3F_8 in comparison with **b)** the Raman spectrum of PbF_2. The calculated Raman intensities are shown in red (DFT-PBE0/TZVP).

The low frequency section of the Raman spectra of Pb_3F_8 and PbF_2 are given in Figure S 7. The Raman bands of both compounds display a similar shape and are distinctly broadened in this frequency range. It is thus difficult to estimate the phase purity of Pb_3F_8 from Raman data as the broad background of the Pb_3F_8 Raman spectrum could result from PbF_2 that is also present as evidenced by powder X-ray diffraction data (see Section Powder X-Ray Diffraction). Pb_3F_8 is best identified by the lattice vibrational bands around 100 cm^{-1} as this frequency region corresponds to a minimum in Raman intensity in the spectrum of PbF_2. Despite the broad background, the maxima of the Pb_3F_8 are well reproduced by our DFT calculations: This allows us to assign the two peaks at around 250 cm^{-1} and the two peaks at around 200 cm^{-1} to a symmetric stretching of the Pb(II)−F bonds and a bending of the Pb(IV)−F bonds, respectively.

Table S 4. Observed bands in the measured Raman spectra of Pb_3F_8 in comparison to PbF_2.

Pb_3F_8		PbF_2	
Raman active mode / cm^{-1}	Assignment	Raman active mode / cm^{-1}	Assignment
531	Pb(IV)−F symmetric stretching	244	Stretching/Bending of the whole lattice
258	Pb(II)−F symmetric stretching + Pb(IV) bending	183	Pb−F asymmetric stretching
241	Pb(II)−F symmetric stretching + Pb(IV) bending	133	Bending of the whole lattice
209	Pb(IV) bending	99	Pb−F wagging/scratching
189	Pb(IV) bending	69	Pb−F scissoring
112	Pb−F bending of the whole lattice		
98	Pb−F bending of the whole lattice		
80	Pb−F bending of the whole lattice		
69	Pb−F bending of the whole lattice		

Table S 5. Band assignment for the calculated Raman spectrum of Pb_3F_8 (DFT-PBE0/TZVP). The calculations were performed in the space group *I*2/*a* (15) with the point group symmetry C_{2h} at the Γ-point.

Frequency / cm^{-1}	IR intensity (arb. units)	Raman intensity (arb. units)	Assignment	Irreducible rep. (C_{2h})
520	0.0	1000.0	Pb(IV)−F symmetric stretching + Pb(II)−F scissoring	*Ag*
517	12.3	0.0	Pb(IV)−F symmetric stretching + Pb(II)−F scissoring	*Au*
493	580.8	0.0	Pb(IV)−F asymmetric stretching + Pb(II)−F scissoring	*Bu*
490	0.0	15.8	Pb(IV)−F asymmetric stretching + Pb(II)−F scissoring	*Bg*
478	0.0	6.7	Pb(IV)−F asymmetric stretching + Pb(II)−F rocking	*Bg*
470	546.0	0.0	Pb(IV)−F asymmetric stretching + Pb(II)−F rocking	*Bu*
464	0.0	11.2	Pb(IV)−F asymmetric stretching + Pb(II)−F rocking	*Ag*
456	554.3	0.0	Pb(IV)−F asymmetric stretching + Pb(II)−F rocking	*Au*
456	290.3	0.0	Pb(IV)−F asymmetric stretching + Pb(II)−F scissoring	*Au*
406	0.0	1.7	Pb(IV)−F asymmetric stretching + Pb(II)−F rocking/wagging	*Bg*
399	0.0	5.9	Pb(IV)−F asymmetric stretching + Pb(II)−F wagging	*Ag*
385	0.5	0.0	Pb(IV)−F asymmetric stretching + Pb(II)−F twisting	*Bu*
329	35.0	0.0	Pb(II)−F symmetric stretching	*Au*
323	0.0	22.5	Pb(II)−F symmetric stretching + Pb(IV)−F scissoring	*Bg*
320	132.3	0.0	Pb(II)−F wagging + Pb(IV)−F scissoring	*Bu*
295	0.0	37.4	Pb(II)−F asymmetric stretching	*Ag*
284	0.0	46.0	Pb(II)−F wagging	*Ag*
253	0.0	82.9	Pb(II)−F symmetric stretching + Pb(IV)−F wagging	*Bg*
253	0.0	111.6	Pb(II)−F symmetric stretching + Pb(IV)−F scissoring	*Ag*
250	17.9	0.0	Pb(II)−F asymmetric stretching + Pb(IV)−F scissoring	*Au*
247	0.0	0.2	Pb(II)−F scissoring + Pb(IV)−F scissoring	*Bg*
242	340.9	0.0	Pb(II)−F symmetric stretching + Pb(IV)−F scissoring	*Bu*
237	3.8	0.0	Pb(II)−F scissoring + Pb(IV)−F scissoring	*Au*
233	0.0	94.6	Pb(II)−F symmetric stretching + Pb(IV)−F scissoring	*Ag*
225	0.0	7.8	Pb(II)−F asymmetric stretching + Pb(IV)−F wagging	*Bg*
215	78.8	0.0	Pb(II)−F asymmetric stretching + Pb(IV)−F scissoring	*Au*
213	231.5	0.0	Pb(IV)−F wagging + Pb(II)−F asymmetric stretching	*Bu*
212	0.0	26.1	Pb−F bending/stretching of the whole lattice	*Bg*
198	9.7	0.0	Pb(II)−F symmetric stretching + Pb(IV)−F scissoring	*Au*
196	354.5	0.0	Pb(II)−F asymmetric stretching + Pb(IV)−F scissoring	*Bu*
195	0.0	54.0	Pb(IV)−F scissoring	*Ag*
189	0.0	69.3	Pb(IV)−F twisting	*Ag*
186	0.1	0.0	Pb(IV)−F scissoring	*Au*
174	0.0	12.4	Pb(IV)−F rocking	*Bg*
160	0.0	160.4	Pb(IV)−F twisting	*Ag*
154	179.6	0.0	Pb−F bending of the whole lattice	*Bu*
150	1050.0	0.0	Pb−F bending of the whole lattice	*Bu*
145	0.0	13.7	Pb−F bending of the whole lattice	*Bg*
129	0.0	19.4	Pb−F bending of the whole lattice	*Bg*
129	351.2	0.0	Pb(II)−F scissoring	*Au*
121	1.2	0.0	Pb−F bending of the whole lattice	*Bu*
116	0.0	137.9	Pb−F bending of the whole lattice	*Ag*
115	379.7	0.0	Pb(IV)−F rocking/scissoring + Pb(II)−F wagging	*Au*
111	0.0	15.44	Pb−F bending of the whole lattice	*Bg*
108	32.9	0.0	Pb−F bending of the whole lattice	*Bu*
105	0.0	29.4	Pb−F bending of the whole lattice	*Ag*
104	36.1	0.0	Pb−F bending of the whole lattice	*Au*
91	0.0	26.8	Pb−F bending of the whole lattice	*Bg*
91	0.0	39.4	Pb−F bending of the whole lattice	*Ag*
90	0.9	0.0	Pb(IV)−F twisting	*Au*
86	51.2	0.0	Pb(IV)−F rocking	*Bu*
82	0.0	25.6	Pb−F bending of the whole lattice	*Ag*
80	0.0	30.3	Pb−F bending/stretching of the whole lattice	*Bg*
76	185.3	0.0	Pb−F bending of the whole lattice	*Au*
74	184.8	0.0	Pb−F bending of the whole lattice	*Bu*
67	0.0	35.0	Pb−F bending of the whole lattice	*Bg*
62	147.1	0.0	Pb−F bending of the whole lattice	*Bu*
59	0.0	2.3	Pb−F bending/stretching of the whole lattice	*Ag*
51	95.3	0.0	Pb−F bending of the whole lattice	*Bu*
51	0.0	25.0	Pb−F bending of the whole lattice	*Bg*
49	0.1	0.0	Pb(II)−F scissoring	*Au*
42	0.0	33.3	Pb−F bending of the whole lattice	*Bg*
35	0.0	115.0	Pb−F bending of the whole lattice	*Ag*
0	0.0	0.0	lattice vibrations (low frequency modes spanning the whole lattice)	*Bu*
0	0.0	0.0	lattice vibrations (low frequency modes spanning the whole lattice)	*Bu*
0	0.0	0.0	lattice vibrations (low frequency modes spanning the whole lattice)	*Au*

Table S 6. Band assignment for the calculated Raman spectrum of PbF_2 (DFT-PBE0/TZVP). The calculations were performed in the space group *Pnma* (62) with the point group symmetry D_{2h} at the Γ-point.

Frequency / cm^{-1}	IR intensity (arb. units)	Raman intensity (arb. Units)	Assignment	Irreducible rep. (D_{2h})
362	3.9	0.0	Stretching/Bending of the whole lattice	*B1u*
326	105.2	0.0	Stretching/Bending of the whole lattice	*B2u*
294	0.0	0.2	Stretching/Bending of the whole lattice	*B3g*
260	0.0	59.5	Pb−F asymmetric stretching	*B3g*
248	0.0	337.8	Pb−F symmetric stretching / scissoring	*Ag*
246	0.0	182.4	Pb−F scissoring	*B2g*
228	140.1	0.0	Pb−F rocking	*B3u*
227	0.0	204.3	Pb−F scissoring	*B1g*
227	0.0	491.9	Pb−F asymmetric stretching	*Ag*
221	57.2	0.0	Pb−F asymmetric stretching	*B1u*
205	0.0	215.6	Pb−F asymmetric stretching + scissoring	*B3g*
204	91.6	0.0	Pb−F asymmetric stretching + rocking	*B2u*
196	0.0	0.0	Pb−F asymmetric stretching + wagging	*Au*
191	0.0	1000.0	Pb−F asymmetric stretching	*Ag*
176	0.0	352.0	Pb−F scissoring	*B2g*
174	143.0	0.0	Pb−F asymmetric stretching + rocking	*B2u*
164	832.1	0.0	Bending of the whole lattice	*B1u*
162	0.0	56.8	Bending of the whole lattice	*B3g*
160	0.0	257.4	Bending of the whole lattice	*B1g*
130	0.0	59.3	Bending of the whole lattice	*Au*
130	0.0	0.0	Pb−F twisting/stretching	*Ag*
124	0.0	141.2	Stretching/Bending of the whole lattice	*B3g*
117	0.0	0.5	Stretching/Bending of the whole lattice	*B3g*
115	100.8	0.0	Stretching/Bending of the whole lattice	*B1u*
113	727.5	0.0	Pb−F scissoring	*B2u*
97	0.0	189.4	Pb−F wagging/stretching	*Ag*
89	1158.8	0.0	Pb−F scissoring	*B3u*
63	0.0	699.5	Pb−F scissoring	*Ag*
61	314.1	0.0	Stretching/Bending of the whole lattice	*B2u*
51	0.0	204.6	Rocking of the Pb atoms	*B1g*
50	19.1	0.0	Bending of the whole lattice	*B1u*
47	0.0	97.2	Rocking of the Pb atoms	*B2g*
27	0.0	0.0	Scissoring of the Pb atoms	*Au*
0	0.0	0.0	lattice vibrations (low frequency modes spanning the whole lattice)	*B3u*
0	0.0	0.0	lattice vibrations (low frequency modes spanning the whole lattice)	*B1u*
0	0.0	0.0	lattice vibrations (low frequency modes spanning the whole lattice)	*B2u*

IR spectroscopy

The IR spectrum was recorded inside a glovebox (MBraun) under argon atmosphere on a Bruker alpha FT-IR spectrometer using the ATR Diamond module with a resolution of 4 cm^{-1}. The spectra were processed with the OPUS software package.[8]

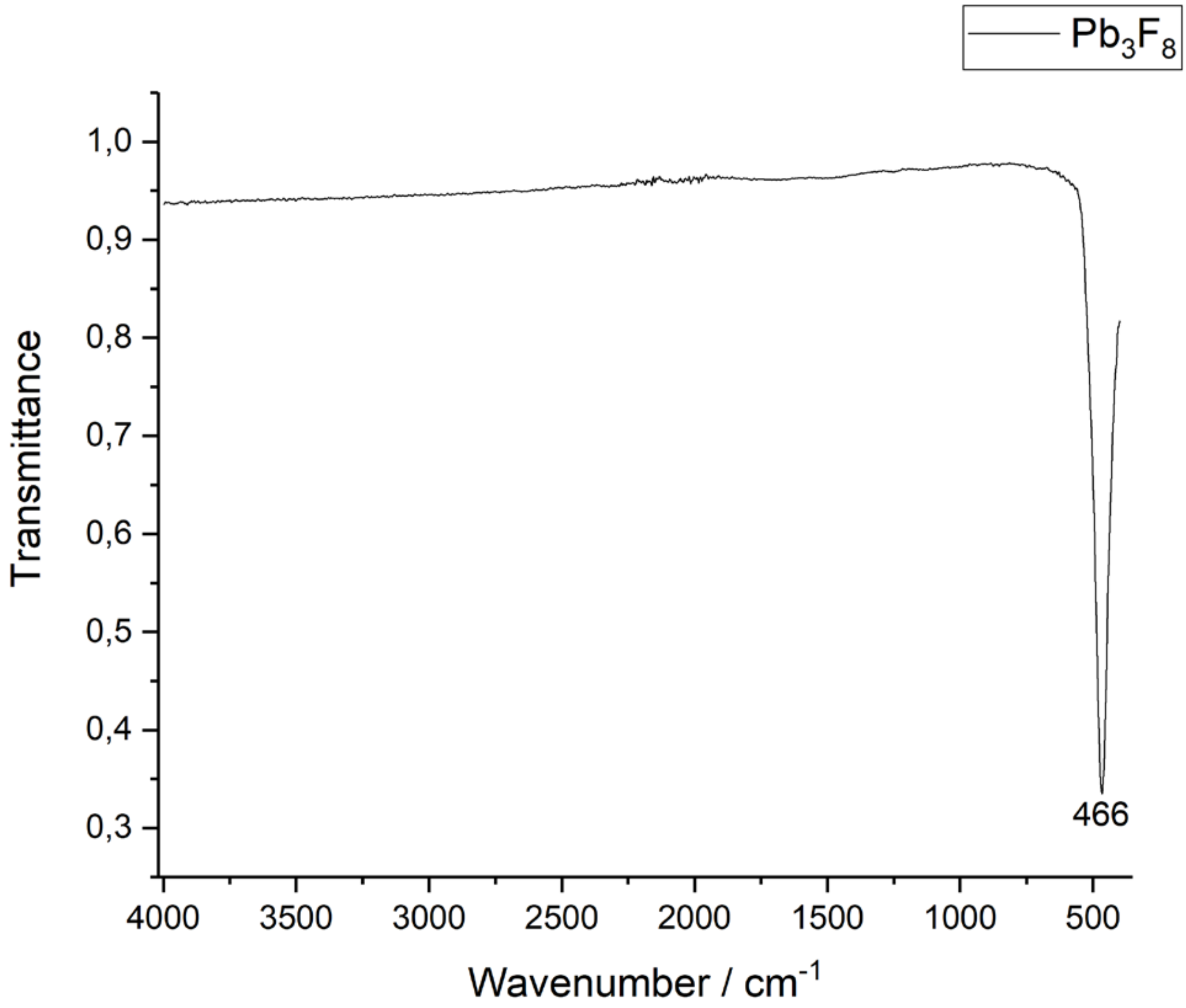

Figure S 8. ATR-IR spectrum of Pb_3F_8.

Solid-state NMR spectroscopy

^{19}F solid-state MAS NMR measurements were performed at 7.05 T on a Bruker Avance II NMR spectrometer at a ^{19}F frequency of 282.406 MHz equipped with a 2.5 mm MAS Bruker double-resonance probe head. The chemical shift of ^{19}F is reported relative to $CFCl_3$. The ^{1}H resonance of 1% TMS in $CDCl_3$ served as an external secondary reference using the Ξ values for ^{19}F as reported by IUPAC.[9,10] ^{19}F MAS NMR spectra were acquired at 20 and 17 kHz spinning rate width a 90° pulse of 2.5 µs and a recycle delay of 4 s. The ^{19}F DEPTH[11,12] NMR spectrum was acquired at 20 kHz spinning rate with 90° and 180° pulses of 2.5 and 5 µs, respectively, and a recycle delay of 4 s. The ^{19}F NMR simulation was performed using SIMPSON-3.1.2.[13]

XPS, HAXPES and NEXAFS

X-ray photoelectron spectroscopy (XPS) was performed with monochromatic Al K_α radiation using a SPECS XR 50 M X-ray anode, a SPECS FOCUS 500 monochromator, and a SPECS PHOIBOS 150 electron energy analyzer equipped with an MCD-9 multi channeltron detector. The spectrometer is housed in a UHV system with a base pressure of $2 \cdot 10^{-10}$ mbar. The sample was prepared in a glove box by evenly distributing a small amount of powder on carbon tape, which was attached to a sample plate. The sample was then transferred into the UHV chamber using a glove bag filled with nitrogen gas to avoid any contact with air. The valence band and the survey spectrum were shifted with the Pb $4f_{7/2}$ peak as reference[14] located at a binding energy of 139.2 eV to correct for charging. For better comparison of the valence region with the DFT calculations, a Shirley background was subtracted.

The near-edge X-ray absorption fine structure (NEXAFS) and hard X-ray photoelectron spectroscopy (HAXPES) measurements were performed at the KMC-1 beamline of the synchrotron radiation facility BESSY II (Helmholtz-Zentrum für Materialien und Energie, Berlin, Germany) using the HIKE end-station with a Scienta R4000 hemispherical electron energy analyzer for HAXPES and a Bruker XFlash 4010 fluorescence detector for NEXAFS. The general properties of this setup are described elsewhere.[15,16] The spot size of the photon beam was approximately 0.3×0.4 mm^2. Typical photon fluxes were in the order of 10^{11} to 10^{12} photons/s over the entire energy range. For sample preparation, a small amount of the pulverulent compounds were distributed on carbon tape (sticking to the sample holder) in such a way that a thin film of the compound was visible to the naked eye. After preparation under ambient conditions, the samples were rapidly transferred to the vacuum to minimize reaction with and contamination by air. The cleanliness of the samples was checked by HAXPES survey spectra. The HAXPES data were referenced to the Au 4f signal of a gold foil located near the sample. The data points of each original spectrum were reduced by a factor of five by averaging five successive points to one point. NEXAFS measurements were performed on the M_5-edge of Pb in the range of 2460 to 2560 eV with a step width of 0.25 eV. For each sample two NEXAFS spectra were recorded, normalized to the ionization current and afterwards averaged. The background was normalized to the same starting and end points.

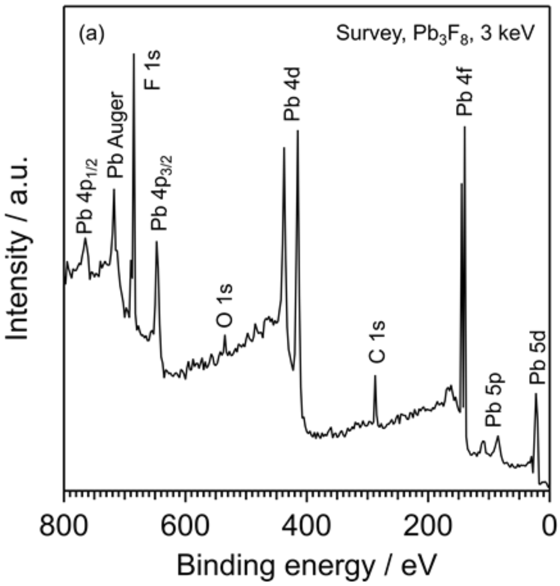

Figure S 9. HAXPES survey spectrum of the Pb_3F_8 sample using a photon energy of 3000 eV. The spectrum shows signs of photoemission-induced charging, as manifested by peak shifts and broadening. The spectrum shows the core levels of Pb and F atoms, besides small C 1s and O 1s contributions from the carbon tape used for mounting the sample. The spectrum confirms that the sample used in the subsequent NEXAFS experiment (see Figure 5c in the main text) is well-defined and unaffected by reaction with ambient air or water vapor.

Table S 7. Assignment of XPS peaks of the Pb_3F_8 survey spectrum in Figure S 9.

Energy / eV	Element	Assignment
21 – 24	Pb 5d	Pb_3F_8
84 – 109	Pb 5p	Pb_3F_8
139 – 145	Pb 4f	Pb_3F_8
286	C 1s	Carbon Tape
415 – 438	Pb 4d	Pb_3F_8
534	O 1s	Carbon Tape
646	Pb $4p_{3/2}$	Pb_3F_8
685	F 1s	Pb_3F_8
718	Pb Auger ($M_4N_{6,7}N_{6,7}$ Transition)	Pb_3F_8
763	Pb $4p_{1/2}$	Pb_3F_8
819	Pb Auger ($M_5N_{6,7}N_{6,7}$ Transition)	Pb_3F_8

Quantum chemical calculations

The vibrational spectra were calculated with the program CRYSTAL17 that uses Gaussian-type atom-centered basis functions[17]. We applied the density functional theory (DFT) PBE0 hybrid functional[18] and triple-zeta-valence + polarization (TZVP) level basis sets for the lead and fluorine atoms. The basis sets were derived from the molecular Karlsruhe basis sets, full basis set details are given below.[19] We applied a 4×4×4 Monkhorst-Pack-type grid of *k*-points for the reciprocal space integration of the electronic structure of Pb_3F_8 and PbF_2. For the evaluation of the Coulomb and exchange integrals (TOLINTEG) we used tightened tolerance factors of 8, 8, 8, 8, and 16. We performed the structural optimizations of the atomic positions and lattice parameters within the constraints imposed by the respective space group symmetry and the default optimization convergence thresholds. The vibrational frequencies were calculated in the harmonic approximation using the data from the structural optimizations.[20,21] The Raman intensities were calculated for a polycrystalline powder sample with total isotropic intensities in arbitrary units adjusting the temperature and laser wavelength to the experimental setup (T = 298.15 K, λ = 533 nm).[22,23] The Raman spectrum was broadened applying a pseudo-Voigt peak profile (50:50 Lorenzian:Gaussian) and a FWHM of 8 cm^{-1}. The peak assignment was carried out by visual inspection of the normal modes (Jmol program package).[24]

We calculated the electronic structure of Pb_3F_8 via DFT using the software package *Quantum Espresso* version 6.3 that is based on plane waves and pseudopotentials.[25] We used scalar as well as full-relativistic norm-conserving pseudopotentials of the SG15 Optimized Norm-Conserving Vanderbilt Pseudopotential Database.[26] We chose the GGA functional PBE and the hybrid functional PBE0 for our calculations.[18,27] The DFT calculations were carried out at the geometry of the experimentally determined single crystal structure. The calculations were performed with an 80 Ry kinetic-energy cutoff, a 320 Ry charge-density cutoff and a centered 4×4×3 Monkhorst-Pack-type grid of *k*-points. In case of the hybrid PBE0 functional, the Fock operator was sampled *via* a Γ-centered grid at each *k*-point. The divergence of the Coulomb potential was treated with the Gygi-Baldereschi approach.[28] We analyzed the electronic structure constructing a set of maximally localized Wannier functions (MLWFs) from the converged ground state electronic density of the plane-wave calculations. We used the program *Wannier90* for this purpose.[29] We defined a target dimension consisting of a set of three *p* orbitals for each fluorine atom and a set of one *s* orbital for each lead atom. The MLWFs were constructed using a 4×4×3 grid of *k*-points. The MLWF basis received can reproduce the F 2*p*/Pb 6*s* valence band as well as the Pb 6*s* conduction band of the plane-wave

electronic structure calculations as shown in Figure S 10. We used the WF basis to calculate Wannier-interpolated band structures as well as densities of states (DOS) with a fine *k*-point sampling of 20×20×20.

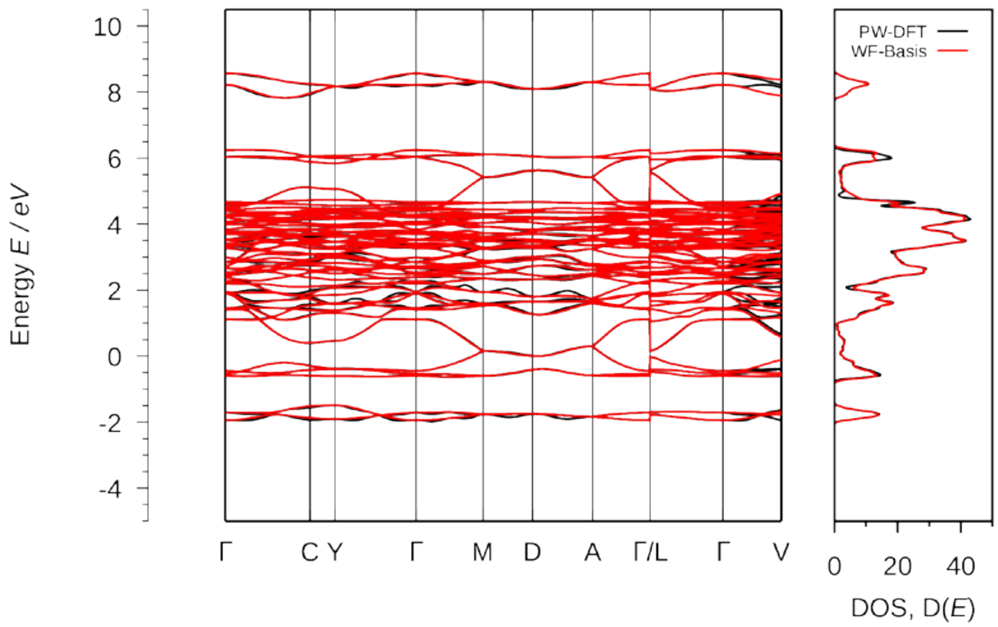

Figure S 10. Left: Electronic band structure of Pb_3F_8. Right: Total Density of States (DOS). Solid red lines: Wannier-interpolated bands. Solid black lines: bands from DFT calculation (DFT-PBE/NCPP).

The obtained projected densities of states were compared to experimentally determined valence band X-ray photoelectron (XP) spectra. The intensities of the calculated pDOSs were adjusted to the experimental data by weighting their intensities with the photoelectron cross sections[30] of the corresponding F 2p and Pb 6s orbitals at the photon energy of 1486.6 eV of the Al K_α radiation and subtracting a Shirley background from the experimental data.

Basis set details for the CRYSTAL17 calculations

F: The TZVP level basis set was taken from a previous study.[31]

Pb: The def2-TZVP basis set with a 60-electron effective core potential was used as a starting point.[19] We fixed the exponents of the outermost s and p functions to 0.09 and reoptimized the exponents of the other s and p functions in the valence space for the lead atom in its ground state. Finally, the outermost s and p functions were combined into one sp-type function. The resulting energy loss with respect to the original molecular basis set is 4.4 mH. The exponent of the outermost d-function was increased from 0.115 to 0.141. The energy cost of this change was only 0.03 mH. The steep f-type polarization function with an exponent of 1.0 was removed. The final basis set in CRYSTAL input format is as follows:

```
282 13
INPUT
22. 0 2 4 4 2 2
12.296303 281.285499 0
8.632634   62.520217 0
10.241790  72.276897 0
8.924176  144.591083 0
6.581342    4.758693 0
6.255403    9.940621 0
7.754336   35.848507 0
7.720281   53.724342 0
4.970264   10.115256 0
4.563789   14.833731 0
3.887512   12.209892 0
3.811963   16.190291 0
5.691577   -9.096665 0
5.715567  -11.531996 0
0 0 4 2.0 1.0
  591.61124370      0.22126521076E-03
  46.757232559      0.56961959130E-02
  20.746462696     -0.21374063831
  14.610796419      0.40502620616
0 0 2 2.0 1.0
  20.181581827     -0.83541883299E-01
  6.4652701641      0.97910892388
0 0 1 0.0 1.0
 1.6683946428       1.0000000000
0 0 1 0.0 1.0
 0.80992082883      1.0000000000
0 0 1 0.0 1.0
 0.23931594794      1.0000000000
0 1 1 0.0 1.0
 0.09               1.0 1.0
0 2 3 6.0 1.0
  15.189102118      0.61952303583
  14.693144415     -0.72498497086
  6.8705890048      0.37680007984
0 2 3 2.0 1.0
  2.2028784073      0.40196284806
  1.2204723142      0.46058131862
 0.63442046718      0.19367655397
0 2 1 0.0 1.0
  0.28121361746     1.0000000000
0 3 6 10.0 1.0
  61.315369628      0.33870800787E-03
  12.372195840      0.13788683942E-01
  6.9254944983     -0.75979608103E-01
  2.3319539939      0.28113784298
  1.2108730003      0.44474512269
 0.60090478506      0.35326874351
0 3 1 0.0 1.0
 0.28135869813      1.0000000000
0 3 1 0.0 1.0
 0.140679349065     1.0000000000
0 4 1 0.0 1.0
 0.28962            1.0000000000
```

Structural Optimization

We have optimized the crystal structure of Pb_3F_8 and PbF_2 with DFT using the hybrid functional PBE0. The results of the optimization are collected in Table S 8 to Table S 11. Overall, the structural data agrees well with the experimental results. The lattice parameters and the volume of the unit cell are overestimated by DFT by 1 % to 2 %. This is probably due to the negligence of relativistic effects in the calculations that should result in a contraction of the Pb 6s orbitals. This also effects the atomic distances that are overestimated by approximately 2 pm by theory. In case of Pb_3F_8 the calculated Pb(IV)−F distances range from 2.080 Å to 2.088 Å compared to experimental distances ranging from 2.048(3) to 2.063(3) Å. In case of Pb(II) DFT yield Pb(IV)−F distances of 2.359 Å to 2.632 Å compared to 2.330(3) to 2.651(3) Å from experiment.

Table S 8. Comparison of experimental cell parameters of Pb_3F_8 (*I*2/*a*, *mS*44) at 100 K with the results of DFT structure optimization (DFT-PBE0/TZVP) at 0 K

	Experiment	DFT	Difference Δ / %
a / Å	8.782(2)	8.89	+1.2
b / Å	7.495(2)	7.56	+0.9
c / Å	10.192(5)	10.33	+1.0
ß / °	98.76(3)	98.40	−0.4
V / Å^3	673.77(8)	686.82	+1.9

Table S 9. Comparison of experimental atomic positions in fractional coordinates of Pb_3F_8 (*I*2/*a*, *mS*44) at 100 K with the results of DFT structure optimization (DFT-PBE0/TZVP) at 0 K.

Atom	Wyckoff	Site sym.		*x*	*y*	*z*
Pb(1)	4*e*	2	Exp.	1/4	0.58692(4)	1/2
			DFT	1/4	0.587	1/2
Pb(2)	8*f*	1	Exp.	0.41214(2)	0.07912(2)	0.65692(2)
			DFT	0.410	0.078	0.656
F(1)	8*f*	1	Exp.	0.2241(4)	0.3868(4)	0.3601(3)
			DFT	0.223	0.385	0.361
F(2)	8*f*	1	Exp.	0.3622(3)	0.0394(4)	0.4277(3)
			DFT	0.362	0.035	0.428
F(3)	8*f*	1	Exp.	0.0579(4)	0.6351(4)	0.5859(3)
			DFT	0.059	0.634	0.590
F(4)	8*f*	1	Exp.	0.3669(4)	0.7490(4)	0.6452(3)
			DFT	0.369	0.748	0.644

Table S 10. Comparison of experimental cell parameters of PbF_2 (*Pnma*, *oP*12) at 293 K with the results of DFT structure optimization (DFT-PBE0/TZVP) at 0 K.

	Experiment	DFT	Difference Δ / %
a / Å	6.4567(1)	6.484	+0.4
b / Å	3.9071(5)	3.909	+0.1
c / Å	7.666(1)	7.743	+1.0
V / $Å^3$	193.39	196.24	+1.5

Table S 11. Comparison of experimental atomic positions in fractional coordinates of PbF_2 (*Pnma*, *oP*12) at 298 K with the results of DFT structure optimization (DFT-PBE0/TZVP) at 0 K.

Atom	Wyckoff	Site sym.		*x*	*y*	*z*
Pb	4*c*	*.m.*	Exp.	0.246(3)	1/4	0.3933(5)
			DFT	0.243	1/4	0.400
F(1)	4*c*	*.m.*	Exp.	0.109(7)	1/4	0.051(7)
			DFT	0.141	1/4	0.067
F(2)	4*c*	*.m.*	Exp.	0.011(8)	1/4	0.650(6)
			DFT	0.021	1/4	0.648

Optimized geometries in CRYSTAL input format

The optimized geometries of Pb_3F_8 in CRYSTAL input format:

```
Pb3F8 P21/c
CRYSTAL
0 0 0
14
5.43382754 5.45042112 13.49734191 110.601641
6
282 3.544324421267E-01  3.552484713783E-02  3.371459639870E-01
282 0.0000 0.0000 0.0000
9   -2.192906516313E-03 -4.814423038974E-01  3.449861753130E-01
9    2.255261635756E-01 -3.166917143964E-01  3.938616342148E-02
9    4.127833958637E-01  3.901304742516E-01  2.579705030519E-01
9    3.376138871129E-01  2.062908644521E-01  3.119245407245E-02
```

The optimized geometries of PbF_2 in CRYSTAL input format:

```
PbF2 Pnma
CRYSTAL
0 0 0
62
6.48416408 3.90862526 7.74313041
3
282 2.438503876734E-01  2.500000000000E-01  4.001980726789E-01
9   2.083538578373E-02  2.500000000000E-01 -3.519218410496E-01
9   1.410241797137E-01  2.500000000000E-01  6.733279160078E-02
```

Literature

[1] *WinXPOW*, STOE & Cie GmbH, Hilpertstrasse 10, 64295 Darmstadt, Germany, **2015**.

[2] A. A. Coelho, *J. Appl. Crystallogr.* **2018**, *51*, 210–218.

[3] *X-Area*, STOE & Cie GmbH, Darmstadt, Germany, **2018**.

[4] G. M. Sheldrick, *Acta Crystallogr., Sect. A: Found. Adv.* **2015**, *71*, 3–8.

[5] G. M. Sheldrick, *Acta Crystallogr., Sect. C: Struct. Chem.* **2015**, *71*, 3–8.

[6] *OriginPro 2017*, OriginLAb Corporation, **2017**.

[7] M. Nespolo, B. Guillot, *J. Appl. Crystallogr.* **2016**, *49*, 317–321.

[8] *OPUS V7.2*, Bruker Optik GmbH, Ettlingen, Germany, **2012**.

[9] R. K. Harris, E. D. Becker, *Journal of Magnetic Resonance* **2002**, *156*, 323–326.

[10] R. K. Harris, E. D. Becker, *Journal of Magnetic Resonance* **2002**, *156*, 323–326.

[11] M. Robin Bendall, R. E. Gordon, *Journal of Magnetic Resonance (1969)* **1983**, *53*, 365–385.

[12] Y. S. Avadhut, J. Weber, E. Hammarberg, C. Feldmann, I. Schellenberg, R. Pöttgen, J. Schmedt auf der Günne, *Chemistry of Materials* **2011**, 110222062928031.

[13] M. Bak, J. T. Rasmussen, N. C. Nielsen, *J. Magn. Reson.* **2000**, *147*, 296–330.

[14] W. E. Morgan, J. R. Van Wazer, *J. Phys. Chem.* **1973**, *77*, 964–969.

[15] F. Schaefers, M. Mertin, M. Gorgoi, *Rev. Sci. Instrum.* **2007**, *78*, 123102.

[16] M. Gorgoi, S. Svensson, F. Schäfers, G. Öhrwall, M. Mertin, P. Bressler, O. Karis, H. Siegbahn, A. Sandell, H. Rensmo, et al., *Nucl. Instruments Methods Phys. Res. Sect. A Accel. Spectrometers, Detect. Assoc. Equip.* **2009**, *601*, 48–53.

[17] R. Dovesi, A. Erba, R. Orlando, C. M. Zicovich-Wilson, B. Civalleri, L. Maschio, M. Rérat, S. Casassa, J. Baima, S. Salustro, et al., *WIREs Comput. Mol. Sci.* **2018**, *8*, 1–36.

[18] C. Adamo, V. Barone, *J. Chem. Phys.* **1999**, *110*, 6158–6170.

[19] F. Weigend, R. Ahlrichs, *Phys. Chem. Chem. Phys.* **2005**, *7*, 3297–3305.

[20] F. Pascale, C. M. Zicovich-Wilson, F. López Gejo, B. Civalleri, R. Orlando, R. Dovesi, *J. Comput. Chem.* **2004**, *25*, 888–897.

[21] C. M. Zicovich-Wilson, F. Pascale, C. Roetti, V. R. Saunders, R. Orlando, R. Dovesi, *J. Comput. Chem.* **2004**, *25*, 1873–1881.

[22] L. Maschio, B. Kirtman, M. Rérat, R. Orlando, R. Dovesi, *J. Chem. Phys.* **2013**, *139*, 164101.

[23] L. Maschio, B. Kirtman, M. Rérat, R. Orlando, R. Dovesi, *J. Chem. Phys.* **2013**, *139*, 164102.

[24] *Jmol: An Open-Source Java Viewer for Chemical Structures in 3D. Http://Www.Jmol.Org/*, Jmol Team, **2017**.

[25] P. Giannozzi et al, *Journal of Physics: Condensed Matter* **2009**, *21*, 1–19.

[26] M. Schlipf, F. Gygi, *Computer Physics Communications* **2015**, *196*, 36–44.

[27] J. P. Perdew, K. Burke, M. Ernzerhof, *Phys. Rev. Lett.* **1996**, *77*, 3865–3868.

[28] F. Gygi, A. Baldereschi, *Phys. Rev. B* **1986**, *34*, 4405–4408.

[29] A. A. Mostofi, J. R. Yates, G. Pizzi, Y.-S. Lee, I. Souza, D. Vanderbilt, N. Marzari, *Computer Physics Communications* **2014**, *185*, 2309–2310.

[30] J. J. Yeh, I. Lindau, *Atomic Data and Nuclear Data Tables* **1985**, *32*, 1–155.

[31] A. J. Karttunen, T. Tynell, M. Karppinen, *J. Phys. Chem. C* **2015**, *119*, 13105–13114.
